# Diethyl [(2,5-di­iodo-4-methyl­phen­yl)meth­yl]phospho­nate

**DOI:** 10.1107/S2414314621006544

**Published:** 2021-06-30

**Authors:** Dieter Schollmeyer, Oleg Sadovski, Heiner Detert

**Affiliations:** a University of Mainz, Institut for Organic Chemistry, Duesbergweg 10-14, 55099 Mainz, Germany; Goethe-Universität Frankfurt, Germany

**Keywords:** crystal structure, iodine, phospho­nate, two-component twin

## Abstract

The title compound, C_12_H_17_I_2_O_3_P, was prepared in three steps from *p*-xylene. Heterodimers between nearly identical mol­ecules are connected *via* three hydrogen bonds from benzylic and ester methyl­ene groups to phospho­nate. The dimers form chains along the *a*-axis direction, stabilized by C—H⋯O bridges.

## Abstract 2

The title compound was prepared in three steps from *p*-xylene. Heterodimers between nearly identical mol­ecules are connected *via* three hydrogen bonds from benzylic and ester methyl­ene groups to phospho­nate. The dimers form chains along the *a*-axis direction, stabilized by C—H⋯O bridges.

## Graphical abstract

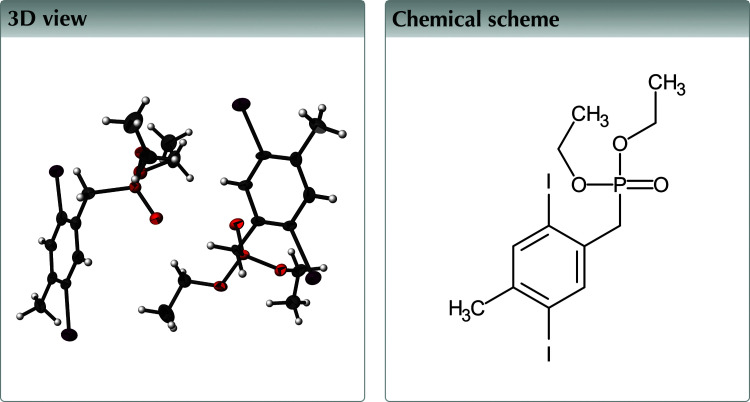

## Structure description

**Figure 1:**
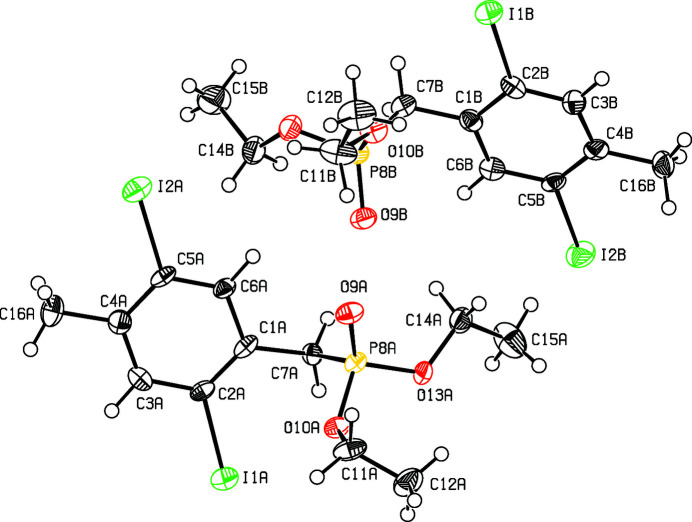
View of the two independent molecules in the title compound. Displacement ellipsoids are drawn at the 50% probability level.

**Figure 2:**
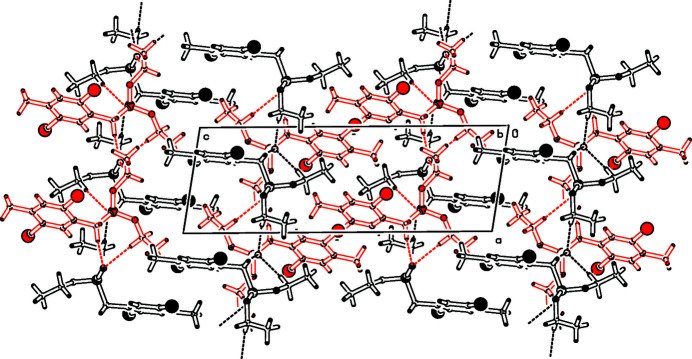
Part of the packing diagram. View along the *b* axis. Hydrogen bonds are drawn with dashed lines. The two independent mol­ecules are drawn in different colours.

In a project focusing on phenyl­ene­vinyl­ene emissive materials (Sugioni & Detert, 2004 ▸; Schmitt *et al.*, 2008 ▸, 2013 ▸) and their electrical and magnetic properties (Cambré *et al.*, 2007 ▸; Nemkovich *et al.*, 2010 ▸), the title compound was prepared as an inter­mediate for fluoro­phores with an *E*-type delayed emission and has been used for the synthesis of π-conjugated cruciforms (Zucchero *et al.*, 2006 ▸). The asymmetric unit contains four mol­ecules. Heterodimers are formed *via* C—H⋯O hydrogen bonds (Table 1 ▸) between nearly identical mol­ecules *A* and *B*. The only significant difference between *A* and *B* (Fig. 1 ▸) is the conformation of one eth­oxy group. The aromatic unit with its four substituents is nearly perfectly planar, with a maximum deviation from the mean plane of 0.013 (16) Å at C3*A*. The bond angles on the 1,2,4,5-tetra­substituted ring nearly match the ideal 120°, only the arene-methyl­ene bond is slightly bent [125.6 (12)° *A*, 123.4(13°) *B*]. The *A*,*B* dimers are connected *via* three slightly bent C—H⋯O hydrogen bridges: C7*A*—H7*A*⋯O9*B* [3.275 (15) Å, 154.3°], C14*A*—H14*B*⋯O9*B* [3.407 (18) Å, 172.2°], and C11*B*—H11*D*⋯O9*A* [3.575 (18) Å, 168.5°]. Three further C—H⋯O bridges connect neighbouring dimers to form chains along the *a*-axis direction (Fig. 2 ▸): C11*A*—H11*A*⋯O9*B* [3.377 (17) Å 157.9°, *B* shifted −1 along *a*], C7*B*—H7*C*⋯O9*A* [3.388 (15) Å, 151.6°, *A* shifted +1 along *a*] and C14*B*—H14*D*⋯O9*A* [3.324 (19) Å, 165.6°, *A* shifted +1 along *a*].

## Synthesis and crystallization

The title compound was prepared from *p*-xylene *via* iodination according to Wirth *et al.* (1964 ▸) and bromination (Wheland & Martin, 1975 ▸) followed by Michaelis–Arbusov reaction. Purification was *via* column chromatography on silica with an eluent gradient. Starting with toluene/ethyl acetate 1/1, the polarity was increased by reducing the toluene concentration first and addition of increasing amounts of methanol. A mixture of the di­iodo compound and some bromo-iodo analogues was obtained. The title compound crystallized from the oily product mixture within 12 years.

## Refinement

Crystal data, data collection and structure refinement details are summarized in Table 2 ▸. The crystal was a two component twin. The fractional contribution of the major domain refined to 0.5155 (14).

## Supplementary Material

Crystal structure: contains datablock(s) I, global. DOI: 10.1107/S2414314621006544/bt4116sup1.cif

Structure factors: contains datablock(s) I. DOI: 10.1107/S2414314621006544/bt4116Isup2.hkl

Supporting information file. DOI: 10.1107/S2414314621006544/bt4116Isup3.cml

CCDC reference: 2091488

Additional supporting information:  crystallographic information; 3D view; checkCIF report

## Figures and Tables

**Table 1:** Hydrogen-bond geometry (Å, °)

*D*—H⋯*A*	*D*—H	H⋯*A*	*D*⋯*A*	*D*—H⋯*A*
C7*A*—H7*A*⋯O9*B*	0.99	2.36	3.275 (15)	154
C11*A*—H11*B*⋯O9*B* ^i^	0.99	2.44	3.377 (17)	158
C14*A*—H14*B*⋯O9*B*	0.99	2.42	3.407 (18)	172
C7*B*—H7*C*⋯O9*A* ^ii^	0.99	2.48	3.388 (15)	152
C11*B*—H11*D*⋯O9*A*	0.99	2.60	3.575 (18)	169
C14*B*—H14*D*⋯O9*A* ^ii^	0.99	2.36	3.324 (19)	166

Symmetry codes: (i) 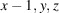 ; (ii) 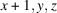 .

**Table 2:** Experimental details

Crystal data	
Chemical formula	C_12_H_17_I_2_O_3_P
*M*_r_	494.02
Crystal system, space group	Triclinic, *P* 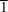
Temperature (K)	120
*a*, *b*, *c* (Å)	7.4909 (6), 10.9201 (9), 20.4720 (17)
α, β, γ (°)	89.070 (7), 80.799 (6), 71.451 (6)
*V* (Å^3^)	1566.1 (2)
*Z*	4
Radiation type	Mo *K*α
μ (mm^−1^)	4.12
Crystal size (mm)	0.11 × 0.10 × 0.04

Data collection	
Diffractometer	Stoe IPDS 2T
Absorption correction	Integration (*X-RED32*; Stoe & Cie, 2019 ▸)
*T*_min_, *T*_max_	0.651, 0.820
No. of measured, independent and observed [*I* > 2σ(*I*)] reflections	22662, 22662, 15127
(sin θ/λ)_max_ (Å^−1^)	0.661

Refinement	
*R*[*F* ^2^ > 2σ(*F* ^2^)], *wR*(*F* ^2^), *S*	0.075, 0.186, 1.09
No. of reflections	22662
No. of parameters	332
H-atom treatment	H-atom parameters constrained
Δρ_max_, Δρ_min_ (e Å^−3^)	1.69, −1.26

Computer programs: *X-AREA WinXpose*, *Recipe* and *Integrate* (Stoe & Cie, 2019 ▸), *SHELXT2014* (Sheldrick, 2015*a* ▸), *SHELXL2018/3* (Sheldrick, 2015*b* ▸) and *PLATON* (Spek, 2020 ▸).

## full crystallographic data

### Crystal data

**Table:**

C_12_H_17_I_2_O_3_P	*Z* = 4
*M**_r_* = 494.02	*F*(000) = 936
Triclinic, *P*1	*D*_x_ = 2.095 Mg m^−^^3^
*a* = 7.4909 (6) Å	Mo *K*α radiation, λ = 0.71073 Å
*b* = 10.9201 (9) Å	Cell parameters from 14985 reflections
*c* = 20.4720 (17) Å	θ = 2.9–28.3°
α = 89.070 (7)°	µ = 4.12 mm^−^^1^
β = 80.799 (6)°	*T* = 120 K
γ = 71.451 (6)°	Column, colourless
*V* = 1566.1 (2) Å^3^	0.11 × 0.10 × 0.04 mm

### Data collection

**Table:**

Stoe IPDS 2T diffractometer	22662 independent reflections
Radiation source: sealed X-ray tube, 12 x 0.4 mm long-fine focus	15127 reflections with *I* > 2σ(*I*)
Detector resolution: 6.67 pixels mm^-1^	θ_max_ = 28.0°, θ_min_ = 2.8°
rotation method scans	*h* = −9→9
Absorption correction: integration (X-Red32; Stoe & Cie, 2019)	*k* = −14→14
*T*_min_ = 0.651, *T*_max_ = 0.820	*l* = −26→26
22662 measured reflections	

### Refinement

**Table:**

Refinement on *F*^2^	Primary atom site location: structure-invariant direct methods
Least-squares matrix: full	Hydrogen site location: inferred from neighbouring sites
*R*[*F*^2^ > 2σ(*F*^2^)] = 0.075	H-atom parameters constrained
*wR*(*F*^2^) = 0.186	*w* = 1/[σ^2^(*F*_o_^2^) + (0.0494*P*)^2^ + 24.353*P*] where *P* = (*F*_o_^2^ + 2*F*_c_^2^)/3
*S* = 1.09	(Δ/σ)_max_ < 0.001
22662 reflections	Δρ_max_ = 1.69 e Å^−^^3^
332 parameters	Δρ_min_ = −1.26 e Å^−^^3^
0 restraints	

### Special details

**Table:**

Geometry. All esds (except the esd in the dihedral angle between two l.s. planes) are estimated using the full covariance matrix. The cell esds are taken into account individually in the estimation of esds in distances, angles and torsion angles; correlations between esds in cell parameters are only used when they are defined by crystal symmetry. An approximate (isotropic) treatment of cell esds is used for estimating esds involving l.s. planes.
Refinement. Hydrogen atoms were placed at calculated positions and were refined in the riding-model approximation with C_aromatic_–H = 0.95 Å, C_methylene_–H = 0.99 Å, C_methyl_–H = 0.98 Å, and with *U*_iso_(H) = 1.2 *U*_eq_(C_aromatic_, C_methylene_) or with *U*_iso_(H) = 1.5 *U*_eq_(C_methyl_). Refined as a 2-component twin. Twin law for transforming hkl(1) to hkl(2): 0.97100 0.03900 0.01800 0.98600 -0.98000 0.00900 0.98600 0.02000 -0.99100

### Fractional atomic coordinates and isotropic or equivalent isotropic displacement parameters (Å^2^)

**Table:**

	*x*	*y*	*z*	*U*_iso_*/*U*_eq_	
I1A	0.75872 (15)	−0.26269 (8)	0.13371 (5)	0.0327 (2)	
I2A	0.68923 (17)	0.33082 (9)	−0.00063 (5)	0.0394 (3)	
C1A	0.6926 (18)	0.0275 (12)	0.1389 (6)	0.023 (3)	
C2A	0.7307 (18)	−0.0795 (11)	0.0976 (6)	0.021 (2)	
C3A	0.749 (2)	−0.0701 (13)	0.0282 (7)	0.028 (3)	
H3A	0.768361	−0.144994	0.001490	0.033*	
C4A	0.739 (2)	0.0447 (13)	−0.0017 (6)	0.027 (3)	
C5A	0.701 (2)	0.1543 (12)	0.0394 (7)	0.028 (3)	
C6A	0.6802 (19)	0.1462 (11)	0.1077 (6)	0.023 (3)	
H6A	0.656970	0.221900	0.134250	0.028*	
C7A	0.6757 (18)	0.0270 (12)	0.2123 (6)	0.022 (2)	
H7A	0.748898	0.080342	0.226169	0.026*	
H7B	0.734980	−0.062605	0.225433	0.026*	
P8A	0.4346 (5)	0.0866 (3)	0.25589 (16)	0.0222 (6)	
O9A	0.3240 (14)	0.2183 (8)	0.2406 (5)	0.030 (2)	
O10A	0.3510 (13)	−0.0234 (8)	0.2415 (4)	0.0245 (19)	
C11A	0.147 (2)	−0.0070 (14)	0.2546 (8)	0.035 (3)	
H11A	0.115876	−0.058664	0.221468	0.042*	
H11B	0.072998	0.084949	0.250701	0.042*	
C12A	0.093 (2)	−0.0489 (17)	0.3216 (8)	0.043 (4)	
H12A	−0.040769	−0.045706	0.328067	0.064*	
H12B	0.110157	0.008676	0.354638	0.064*	
H12C	0.174274	−0.137581	0.326763	0.064*	
O13A	0.4539 (15)	0.0703 (9)	0.3315 (4)	0.028 (2)	
C14A	0.495 (2)	0.1709 (14)	0.3673 (7)	0.031 (3)	
H14A	0.374929	0.242258	0.382349	0.038*	
H14B	0.582833	0.206435	0.337867	0.038*	
C15A	0.583 (3)	0.1122 (17)	0.4254 (9)	0.053 (5)	
H15A	0.486186	0.093330	0.458543	0.080*	
H15B	0.633866	0.172867	0.444834	0.080*	
H15C	0.686594	0.031969	0.411094	0.080*	
C16A	0.764 (2)	0.0499 (15)	−0.0766 (7)	0.034 (3)	
H16A	0.771780	−0.033817	−0.095456	0.051*	
H16B	0.881793	0.069548	−0.093195	0.051*	
H16C	0.654934	0.117414	−0.089395	0.051*	
I1B	0.63866 (15)	0.75758 (9)	0.36179 (5)	0.0338 (2)	
I2B	1.01458 (17)	0.17183 (9)	0.50120 (5)	0.0397 (3)	
C1B	0.868 (2)	0.4691 (13)	0.3608 (7)	0.026 (3)	
C2B	0.752 (2)	0.5771 (13)	0.4004 (7)	0.027 (3)	
C3B	0.713 (2)	0.5666 (13)	0.4682 (6)	0.027 (3)	
H3B	0.635236	0.641517	0.494047	0.032*	
C4B	0.782 (2)	0.4539 (13)	0.5001 (7)	0.026 (3)	
C5B	0.896 (2)	0.3482 (12)	0.4599 (6)	0.024 (3)	
C6B	0.9378 (18)	0.3541 (13)	0.3916 (7)	0.025 (3)	
H6B	1.014923	0.278705	0.365942	0.030*	
C7B	0.9231 (19)	0.4726 (12)	0.2861 (6)	0.026 (3)	
H7C	1.060215	0.423153	0.273632	0.031*	
H7D	0.905885	0.563363	0.274129	0.031*	
P8B	0.7894 (5)	0.4086 (3)	0.23870 (16)	0.0238 (7)	
O9B	0.7928 (15)	0.2762 (8)	0.2537 (5)	0.030 (2)	
O10B	0.5903 (14)	0.5151 (9)	0.2494 (5)	0.031 (2)	
C11B	0.442 (2)	0.5089 (14)	0.2103 (9)	0.040 (4)	
H11C	0.495951	0.496176	0.162590	0.048*	
H11D	0.395726	0.435547	0.224232	0.048*	
C12B	0.282 (2)	0.6327 (16)	0.2225 (10)	0.049 (4)	
H12D	0.332262	0.705147	0.213715	0.073*	
H12E	0.219332	0.639049	0.268695	0.073*	
H12F	0.189932	0.635602	0.193100	0.073*	
O13B	0.8694 (15)	0.4266 (9)	0.1634 (4)	0.030 (2)	
C14B	1.036 (2)	0.3272 (14)	0.1298 (7)	0.035 (3)	
H14C	0.997241	0.264028	0.106133	0.042*	
H14D	1.116079	0.280608	0.162241	0.042*	
C15B	1.146 (3)	0.3913 (18)	0.0819 (10)	0.051 (5)	
H15D	1.256681	0.325726	0.057019	0.076*	
H15E	1.189278	0.450408	0.106048	0.076*	
H15F	1.063975	0.440126	0.051230	0.076*	
C16B	0.736 (2)	0.4469 (16)	0.5744 (7)	0.036 (3)	
H16D	0.684167	0.375599	0.585012	0.053*	
H16E	0.640661	0.528407	0.592605	0.053*	
H16F	0.851940	0.432194	0.593624	0.053*	

### Atomic displacement parameters (Å^2^)

**Table:**

	*U*^11^	*U*^22^	*U*^33^	*U*^12^	*U*^13^	*U*^23^
I1A	0.0344 (5)	0.0278 (4)	0.0367 (5)	−0.0107 (4)	−0.0074 (4)	0.0072 (4)
I2A	0.0430 (6)	0.0349 (5)	0.0403 (5)	−0.0136 (4)	−0.0055 (4)	0.0130 (4)
C1A	0.016 (6)	0.029 (6)	0.025 (6)	−0.008 (5)	−0.005 (5)	0.013 (5)
C2A	0.016 (6)	0.021 (5)	0.028 (6)	−0.005 (5)	−0.005 (5)	0.010 (5)
C3A	0.027 (7)	0.025 (6)	0.031 (6)	−0.007 (5)	−0.007 (5)	−0.006 (5)
C4A	0.025 (7)	0.036 (7)	0.027 (6)	−0.016 (6)	−0.006 (5)	0.001 (6)
C5A	0.032 (8)	0.025 (6)	0.028 (6)	−0.011 (5)	−0.007 (5)	0.015 (5)
C6A	0.025 (6)	0.018 (5)	0.024 (6)	−0.004 (5)	−0.002 (5)	0.006 (5)
C7A	0.024 (6)	0.024 (6)	0.020 (5)	−0.008 (5)	−0.012 (5)	0.003 (5)
P8A	0.0215 (16)	0.0229 (14)	0.0223 (14)	−0.0073 (12)	−0.0043 (12)	0.0062 (12)
O9A	0.026 (5)	0.024 (4)	0.038 (5)	−0.007 (4)	−0.005 (4)	0.008 (4)
O10A	0.019 (4)	0.026 (4)	0.030 (4)	−0.010 (3)	−0.003 (4)	0.004 (4)
C11A	0.025 (7)	0.032 (7)	0.053 (9)	−0.015 (6)	−0.011 (6)	0.015 (6)
C12A	0.041 (9)	0.057 (9)	0.040 (8)	−0.031 (8)	−0.007 (7)	0.013 (7)
O13A	0.040 (6)	0.026 (4)	0.017 (4)	−0.010 (4)	−0.004 (4)	0.004 (4)
C14A	0.034 (8)	0.040 (7)	0.025 (6)	−0.018 (6)	−0.002 (5)	0.002 (6)
C15A	0.056 (11)	0.044 (9)	0.056 (10)	0.001 (8)	−0.031 (9)	−0.007 (8)
C16A	0.026 (7)	0.047 (8)	0.025 (6)	−0.007 (6)	−0.005 (5)	0.005 (6)
I1B	0.0360 (5)	0.0285 (4)	0.0367 (5)	−0.0093 (4)	−0.0080 (4)	0.0068 (4)
I2B	0.0475 (6)	0.0346 (5)	0.0398 (5)	−0.0125 (4)	−0.0172 (4)	0.0130 (4)
C1B	0.024 (7)	0.027 (6)	0.028 (6)	−0.011 (5)	−0.005 (5)	−0.002 (6)
C2B	0.025 (7)	0.025 (6)	0.033 (7)	−0.008 (5)	−0.005 (5)	−0.006 (6)
C3B	0.026 (7)	0.029 (6)	0.027 (6)	−0.008 (5)	−0.009 (5)	0.003 (5)
C4B	0.025 (7)	0.031 (6)	0.031 (6)	−0.019 (6)	−0.008 (5)	0.004 (6)
C5B	0.024 (7)	0.027 (6)	0.028 (6)	−0.017 (5)	−0.009 (5)	0.009 (5)
C6B	0.017 (6)	0.028 (6)	0.033 (6)	−0.008 (5)	−0.007 (5)	0.001 (5)
C7B	0.022 (6)	0.026 (6)	0.030 (6)	−0.008 (5)	−0.005 (5)	0.006 (5)
P8B	0.0235 (17)	0.0233 (14)	0.0260 (15)	−0.0078 (12)	−0.0074 (13)	0.0034 (13)
O9B	0.034 (5)	0.027 (5)	0.033 (5)	−0.014 (4)	−0.011 (4)	0.008 (4)
O10B	0.024 (5)	0.033 (5)	0.037 (5)	−0.009 (4)	−0.009 (4)	0.005 (4)
C11B	0.024 (7)	0.032 (7)	0.070 (10)	−0.009 (6)	−0.021 (7)	0.003 (7)
C12B	0.027 (8)	0.049 (9)	0.073 (11)	−0.014 (7)	−0.017 (8)	0.010 (9)
O13B	0.033 (6)	0.030 (5)	0.025 (4)	−0.007 (4)	−0.001 (4)	0.004 (4)
C14B	0.040 (9)	0.034 (7)	0.026 (6)	−0.006 (6)	−0.003 (6)	0.003 (6)
C15B	0.042 (10)	0.051 (9)	0.059 (10)	−0.023 (8)	0.010 (8)	−0.005 (9)
C16B	0.043 (9)	0.048 (8)	0.024 (6)	−0.025 (7)	−0.008 (6)	−0.001 (6)

### Geometric parameters (Å, º)

**Table:**

I1A—C2A	2.080 (12)	I1B—C2B	2.082 (15)
I2A—C5A	2.064 (13)	I2B—C5B	2.077 (14)
C1A—C2A	1.380 (17)	C1B—C6B	1.38 (2)
C1A—C6A	1.418 (18)	C1B—C2B	1.398 (17)
C1A—C7A	1.487 (16)	C1B—C7B	1.521 (18)
C2A—C3A	1.410 (18)	C2B—C3B	1.382 (19)
C3A—C4A	1.37 (2)	C3B—C4B	1.37 (2)
C3A—H3A	0.9500	C3B—H3B	0.9500
C4A—C5A	1.400 (18)	C4B—C5B	1.385 (18)
C4A—C16A	1.517 (18)	C4B—C16B	1.510 (19)
C5A—C6A	1.386 (18)	C5B—C6B	1.388 (19)
C6A—H6A	0.9500	C6B—H6B	0.9500
C7A—P8A	1.799 (13)	C7B—P8B	1.792 (14)
C7A—H7A	0.9900	C7B—H7C	0.9900
C7A—H7B	0.9900	C7B—H7D	0.9900
P8A—O9A	1.471 (9)	P8B—O9B	1.466 (9)
P8A—O10A	1.572 (9)	P8B—O10B	1.558 (10)
P8A—O13A	1.579 (9)	P8B—O13B	1.595 (10)
O10A—C11A	1.462 (17)	O10B—C11B	1.487 (18)
C11A—C12A	1.47 (2)	C11B—C12B	1.49 (2)
C11A—H11A	0.9900	C11B—H11C	0.9900
C11A—H11B	0.9900	C11B—H11D	0.9900
C12A—H12A	0.9800	C12B—H12D	0.9800
C12A—H12B	0.9800	C12B—H12E	0.9800
C12A—H12C	0.9800	C12B—H12F	0.9800
O13A—C14A	1.465 (16)	O13B—C14B	1.450 (17)
C14A—C15A	1.49 (2)	C14B—C15B	1.49 (2)
C14A—H14A	0.9900	C14B—H14C	0.9900
C14A—H14B	0.9900	C14B—H14D	0.9900
C15A—H15A	0.9800	C15B—H15D	0.9800
C15A—H15B	0.9800	C15B—H15E	0.9800
C15A—H15C	0.9800	C15B—H15F	0.9800
C16A—H16A	0.9800	C16B—H16D	0.9800
C16A—H16B	0.9800	C16B—H16E	0.9800
C16A—H16C	0.9800	C16B—H16F	0.9800
			
C2A—C1A—C6A	116.4 (11)	C6B—C1B—C2B	117.9 (12)
C2A—C1A—C7A	125.6 (12)	C6B—C1B—C7B	118.8 (11)
C6A—C1A—C7A	117.8 (11)	C2B—C1B—C7B	123.4 (13)
C1A—C2A—C3A	121.6 (12)	C3B—C2B—C1B	119.8 (14)
C1A—C2A—I1A	122.2 (9)	C3B—C2B—I1B	117.6 (9)
C3A—C2A—I1A	116.1 (9)	C1B—C2B—I1B	122.6 (10)
C4A—C3A—C2A	121.6 (11)	C4B—C3B—C2B	123.5 (12)
C4A—C3A—H3A	119.2	C4B—C3B—H3B	118.2
C2A—C3A—H3A	119.2	C2B—C3B—H3B	118.2
C3A—C4A—C5A	117.6 (12)	C3B—C4B—C5B	115.7 (12)
C3A—C4A—C16A	119.8 (12)	C3B—C4B—C16B	121.7 (12)
C5A—C4A—C16A	122.6 (13)	C5B—C4B—C16B	122.6 (13)
C6A—C5A—C4A	121.2 (12)	C4B—C5B—C6B	122.8 (13)
C6A—C5A—I2A	118.2 (9)	C4B—C5B—I2B	120.1 (9)
C4A—C5A—I2A	120.6 (10)	C6B—C5B—I2B	117.1 (9)
C5A—C6A—C1A	121.6 (11)	C1B—C6B—C5B	120.4 (12)
C5A—C6A—H6A	119.2	C1B—C6B—H6B	119.8
C1A—C6A—H6A	119.2	C5B—C6B—H6B	119.8
C1A—C7A—P8A	114.5 (9)	C1B—C7B—P8B	114.7 (10)
C1A—C7A—H7A	108.6	C1B—C7B—H7C	108.6
P8A—C7A—H7A	108.6	P8B—C7B—H7C	108.6
C1A—C7A—H7B	108.6	C1B—C7B—H7D	108.6
P8A—C7A—H7B	108.6	P8B—C7B—H7D	108.6
H7A—C7A—H7B	107.6	H7C—C7B—H7D	107.6
O9A—P8A—O10A	116.1 (6)	O9B—P8B—O10B	116.8 (6)
O9A—P8A—O13A	113.4 (5)	O9B—P8B—O13B	114.2 (5)
O10A—P8A—O13A	102.7 (5)	O10B—P8B—O13B	101.9 (6)
O9A—P8A—C7A	115.6 (6)	O9B—P8B—C7B	114.9 (6)
O10A—P8A—C7A	102.7 (5)	O10B—P8B—C7B	102.5 (6)
O13A—P8A—C7A	104.8 (6)	O13B—P8B—C7B	104.8 (6)
C11A—O10A—P8A	123.0 (8)	C11B—O10B—P8B	120.1 (8)
O10A—C11A—C12A	110.5 (13)	C12B—C11B—O10B	107.8 (12)
O10A—C11A—H11A	109.6	C12B—C11B—H11C	110.1
C12A—C11A—H11A	109.6	O10B—C11B—H11C	110.1
O10A—C11A—H11B	109.6	C12B—C11B—H11D	110.1
C12A—C11A—H11B	109.6	O10B—C11B—H11D	110.1
H11A—C11A—H11B	108.1	H11C—C11B—H11D	108.5
C11A—C12A—H12A	109.5	C11B—C12B—H12D	109.5
C11A—C12A—H12B	109.5	C11B—C12B—H12E	109.5
H12A—C12A—H12B	109.5	H12D—C12B—H12E	109.5
C11A—C12A—H12C	109.5	C11B—C12B—H12F	109.5
H12A—C12A—H12C	109.5	H12D—C12B—H12F	109.5
H12B—C12A—H12C	109.5	H12E—C12B—H12F	109.5
C14A—O13A—P8A	119.4 (9)	C14B—O13B—P8B	118.9 (9)
O13A—C14A—C15A	108.4 (13)	O13B—C14B—C15B	107.8 (12)
O13A—C14A—H14A	110.0	O13B—C14B—H14C	110.2
C15A—C14A—H14A	110.0	C15B—C14B—H14C	110.2
O13A—C14A—H14B	110.0	O13B—C14B—H14D	110.2
C15A—C14A—H14B	110.0	C15B—C14B—H14D	110.2
H14A—C14A—H14B	108.4	H14C—C14B—H14D	108.5
C14A—C15A—H15A	109.5	C14B—C15B—H15D	109.5
C14A—C15A—H15B	109.5	C14B—C15B—H15E	109.5
H15A—C15A—H15B	109.5	H15D—C15B—H15E	109.5
C14A—C15A—H15C	109.5	C14B—C15B—H15F	109.5
H15A—C15A—H15C	109.5	H15D—C15B—H15F	109.5
H15B—C15A—H15C	109.5	H15E—C15B—H15F	109.5
C4A—C16A—H16A	109.5	C4B—C16B—H16D	109.5
C4A—C16A—H16B	109.5	C4B—C16B—H16E	109.5
H16A—C16A—H16B	109.5	H16D—C16B—H16E	109.5
C4A—C16A—H16C	109.5	C4B—C16B—H16F	109.5
H16A—C16A—H16C	109.5	H16D—C16B—H16F	109.5
H16B—C16A—H16C	109.5	H16E—C16B—H16F	109.5
			
C6A—C1A—C2A—C3A	−2.4 (19)	C6B—C1B—C2B—C3B	1 (2)
C7A—C1A—C2A—C3A	−178.4 (13)	C7B—C1B—C2B—C3B	−178.3 (13)
C6A—C1A—C2A—I1A	179.0 (10)	C6B—C1B—C2B—I1B	179.4 (10)
C7A—C1A—C2A—I1A	3.0 (18)	C7B—C1B—C2B—I1B	0.2 (19)
C1A—C2A—C3A—C4A	3 (2)	C1B—C2B—C3B—C4B	−1 (2)
I1A—C2A—C3A—C4A	−177.9 (11)	I1B—C2B—C3B—C4B	−179.1 (11)
C2A—C3A—C4A—C5A	−3 (2)	C2B—C3B—C4B—C5B	0 (2)
C2A—C3A—C4A—C16A	178.6 (13)	C2B—C3B—C4B—C16B	179.9 (14)
C3A—C4A—C5A—C6A	2 (2)	C3B—C4B—C5B—C6B	−1 (2)
C16A—C4A—C5A—C6A	−179.6 (14)	C16B—C4B—C5B—C6B	179.8 (13)
C3A—C4A—C5A—I2A	178.7 (11)	C3B—C4B—C5B—I2B	178.6 (10)
C16A—C4A—C5A—I2A	−3.0 (19)	C16B—C4B—C5B—I2B	−0.9 (18)
C4A—C5A—C6A—C1A	−1 (2)	C2B—C1B—C6B—C5B	−1 (2)
I2A—C5A—C6A—C1A	−178.0 (10)	C7B—C1B—C6B—C5B	178.0 (12)
C2A—C1A—C6A—C5A	1 (2)	C4B—C5B—C6B—C1B	1 (2)
C7A—C1A—C6A—C5A	177.7 (13)	I2B—C5B—C6B—C1B	−178.2 (10)
C2A—C1A—C7A—P8A	−104.0 (13)	C6B—C1B—C7B—P8B	79.6 (14)
C6A—C1A—C7A—P8A	80.1 (14)	C2B—C1B—C7B—P8B	−101.3 (14)
C1A—C7A—P8A—O9A	−56.9 (11)	C1B—C7B—P8B—O9B	−55.2 (11)
C1A—C7A—P8A—O10A	70.5 (10)	C1B—C7B—P8B—O10B	72.5 (11)
C1A—C7A—P8A—O13A	177.4 (9)	C1B—C7B—P8B—O13B	178.6 (9)
O9A—P8A—O10A—C11A	−39.4 (12)	O9B—P8B—O10B—C11B	−62.1 (12)
O13A—P8A—O10A—C11A	84.9 (11)	O13B—P8B—O10B—C11B	63.0 (12)
C7A—P8A—O10A—C11A	−166.5 (10)	C7B—P8B—O10B—C11B	171.3 (11)
P8A—O10A—C11A—C12A	−91.8 (13)	P8B—O10B—C11B—C12B	−170.5 (12)
O9A—P8A—O13A—C14A	−42.8 (12)	O9B—P8B—O13B—C14B	−40.2 (13)
O10A—P8A—O13A—C14A	−168.8 (10)	O10B—P8B—O13B—C14B	−167.0 (11)
C7A—P8A—O13A—C14A	84.3 (11)	C7B—P8B—O13B—C14B	86.4 (11)
P8A—O13A—C14A—C15A	−157.0 (12)	P8B—O13B—C14B—C15B	−147.0 (12)

### Hydrogen-bond geometry (Å, º)

**Table:**

*D*—H···*A*	*D*—H	H···*A*	*D*···*A*	*D*—H···*A*
C7*A*—H7*A*···O9*B*	0.99	2.36	3.275 (15)	154
C11*A*—H11*B*···O9*B*^i^	0.99	2.44	3.377 (17)	158
C14*A*—H14*B*···O9*B*	0.99	2.42	3.407 (18)	172
C7*B*—H7*C*···O9*A*^ii^	0.99	2.48	3.388 (15)	152
C11*B*—H11*D*···O9*A*	0.99	2.60	3.575 (18)	169
C14*B*—H14*D*···O9*A*^ii^	0.99	2.36	3.324 (19)	166

Symmetry codes: (i) *x*−1, *y*, *z*; (ii) *x*+1, *y*, *z*.
